# Endometrial tuberculosis among patients undergoing endometrial biopsy at Tikur Anbesa specialized hospital, Addis Ababa, Ethiopia

**DOI:** 10.1186/s12879-018-3202-x

**Published:** 2018-07-05

**Authors:** Sileshi Abdissa, Tamrat Abebe, Gobena Ameni, Sisay Teklu, Yonas Bekuretsion, Markos Abebe, Adane Mihret

**Affiliations:** 1 Arsi University, Asella, Ethiopia; 2 Department of Microbiology, Immunology and Parasitology, School of Medicine, Addis Ababa University, Addis Ababa, Ethiopia; 3 Aklilu Lemma Institute of Pathobiology, Addis Ababa University, Addis Ababa, Ethiopia; 4 Department of Gynecology and Obstetrics, School of Medicine, Addis Ababa University, Addis Ababa, Ethiopia; 5 Department of Pathology, School of Medicine, Addis Ababa University, Addis Ababa, Ethiopia; 6 Armauer Hansen Research Institute, Addis Ababa, Ethiopia

**Keywords:** Endometrium, Tuberculosis, Mycobacterium tuberculosis

## Abstract

**Background:**

Female genital tuberculosis (FGTB) is known to cause severe tubal disease leading to infertility and its incidence closely parallels with the overall prevalence of tuberculosis (TB) in a community. Its magnitude is underreported because diagnosis is difficult and requires invasive techniques. In this study we determined the prevalence of endometrial tuberculosis and characterized isolates among women who underwent endometrial biopsy for evaluation of various conditions at a Tikur Anbessa Specialized Hospital (TAHS), Addis Ababa, Ethiopia.

**Methods:**

A cross sectional study was conducted on 152 consecutive gynecologic patients who underwent endometrial biopsy for different gynecologic diseases. Endometrial tissue taken for routine histopathology examination was shared after informed consent was obtained from the patient and subjected to polymerase chain reaction (PCR) and culture for *Mycobacterium tuberculosis (Mtb)*.

**Results:**

The prevalence of endometrial TB in this study by IS1081PCR was 4.6% (7/152) while culture proven endometrial TB was 2.6% (4/152). However, histological examination identified only 2/152 (1.3%) endometrial tuberculosis. While all culture proven TB samples were also PCR positive for Mtb, only one histologic proven endometrial TB was culture and PCR positive. All of the four isolates by culture were *M. tuberculosis*.

**Conclusion:**

This study has shown that the magnitude of endometrial TB is fairly high in gynecologic patients visiting outpatient departments for various complaints and PCR detects more cases than culture or Histopathology.

## Background

Tuberculosis (TB) predominately presents with pulmonary disease, although extra-pulmonary TB (EPTB) is not uncommon [1]. Genitourinary TB, comprising about 30% of all TB, is the second most common form of extrapulmonary TB [2]. The prevalence of FGTB increases in countries with a high burden of high Pulmonary Tuberculosis (PTB). Five to 13% of all PTB patients develop GTB [3].

In addition to the subtle presentation of the disease, the low sensitivity and specificity of routine diagnostic methods and the paucity of the organism in clinical samples are the main factors for the lower detection of genital TB (GTB) [4]. Therefore, the use of advanced techniques such as PCR as part of diagnosis is crucial in defining the magnitude of the problem and alerting early investigation, before serious complications occur. Most cases of GTB are secondary to gastrointestinal TB spreading to the fallopian tubes causing TB salpingitis. Further spread of the organism involves the uterine endometrium (50%), ovaries (10–30%), cervix (3%), and vagina and vulva (< 1%) [5]. The most frequent symptom of FGTB is infertility, as a result of irreversible damage to the fallopian tube [6]. TB salpingitis and endometritis are responsible for 5 to 20% of all causes of infertility [7, 8], and up to 39–41%in women with tubal factor infertility. Peritubal adhesions and pelvic masses were detected in most patients with GTB [9]. Women with GTB have a low fertility rate varying from 16 to 38.2% [6, 9]. Although most cases of GTB are asymptomatic, chronic pelvic inflammatory disease, menstrual irregularities, low grade fever, loss of weight and appetite, and tubo-ovarian masses are manifestations of GTB [10, 11]. Depending upon the damage to the uterine cavity and the endometrium, uterine tuberculosis can be described as mild, moderate, or severe [9].

Based on the epidemiology of TB and Ethiopia being one of the high TB burden countries, FGTB could also be a common gynecologic problem in Ethiopia. However, there is limited information on the epidemiology of FGTB in general and endometrial TB in particular, in Ethiopia. Studying the concordance of the different laboratory tests with clinical diagnosis is very important to choose the method with better sensitivity. The main purpose of this study was to determine the prevalence of endometrial tuberculosis and to characterize isolates among patients undergoing endometrial biopsy for histo-pathological examination, for gynecologic diseases, at Tikur Anbessa Specialized Hospital, using culture and PCR techniques, and to compare the three methods.

## Methods

### Study design and population

**Fig. 1:**
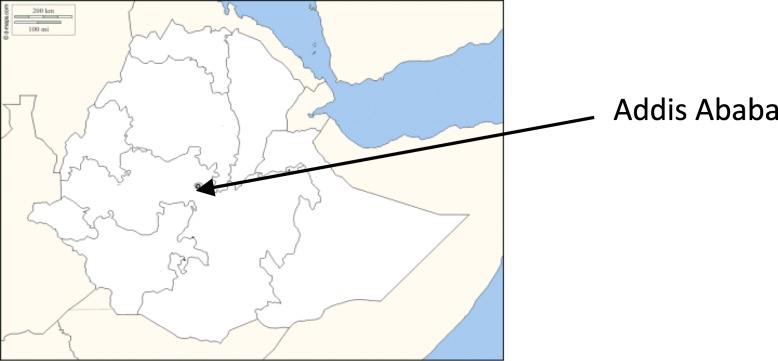
Study area map

An observational cross–sectional, hospital–based study was conducted on 152 women who attended the TASH gynecology outpatient department, Addis Ababa, Ethiopia (Fig. 1). All study participants were gynecologic patients for whom endometrial biopsy was requested for different indications during the period of December 2011–August 2012. All participants gave written informed consent to participate in the study and the study adheres to STROBE guidelines. Fresh, leftover biopsy sample was taken and added aseptically into sterile universal bottles in 5 ml of 0.85% saline solution. It was transported as soon as possible in an ice box at a temperature of 4 °C to laboratory and was processed accordingly.

### Laboratory methods

#### Histopathology

The specimen was transported to the TASH pathology lab in 10% formalin. After gross description by a pathologist, it was transferred into plastic cassettes and processed using Tissue Tek II VIP processor and then embedded in paraffin. After sectioning, it was stained by the Hematoxylin & Eosin (H&E) stain using a Leica autostainer. The Presence of caseating granulomas surrounded by epitheloid cells, lymphocytes, plasma cells and giant cells were diagnostic of tuberculosis**.**

#### Culturing and identification of Mycobacterium

Specimen processing and culturing for isolation of *Mycobacterium tuberculosis* was carried out in the TB laboratory of Akililu Lemma Institute of Patho-Biology (ALIPB) and at the Armauer Hansen Research Institute (AHRI). In the laboratory, the tissue sample was homogenized using 0.5-1 ml sterile saline after sectioning each tissue specimen into fine pieces with a sterile scalpel or scissor. The homogenate was then decontaminated as per Kubica [12] using 4% NaOH for 15 min and centrifuged at 3000 rpm for another 15 min. Two drops of phenol red indicator were added to the sediment after the supernatant was discarded and 2 M HCl was added to neutralize. Neutralization was deemed to be achieved when the color of the solution is changed from purple to yellow. Then the sediment was inoculated immediately onto culture medium and incubated at 37 °C. The incubation period was for at least 8 wks, with weekly observation for discernible growth. Identification was based on morphology, color, rate of growth, and the acid-fastness was confirmed by Ziehl-Neelsen (ZN) staining) [13]. Thereafter, isolates from the positive cultures were preserved by freezing, while at the same time an aliquot was heat–killed ina water bath at 80 °C for 1 h. The frozen and heat–killed isolates were stored at − 20 °C for further mycobacteriology and molecular typing analysis.

#### Region of difference based PCR

Isolates were confirmed as *M. tuberculosis* by deletion typing of the Region of Difference (RD) using 9 regions according to a previously described PCR protocol [14]. The status of the RD9 region (deleted or intact) was assessed by multiplex PCR with a set of three primers (primer set RD9): two primers targeting the flanking regions of RD9 (RD9_Fw, 5′- AAC ACG GTC ACG TTG TCG TG -3′, RD9_Rev, 5′- CAA ACC AGC AGC TGT CGT TG -3’and one primer hybridizing with the internal region of RD9 (RD9_IntRev, 5′- TTG CTT CCC CGG TTC GTC TG -3′. Using agarose gel electrophoresis, a PCR product of 396 bp was interpreted as that RD9 was present (i.e. *M. tuberculosis*). The presence of a 707 bp deletion confirmed *Mycobacterium. africanum* or *Mycobacterium. bovis*.

The PCR mixture used to detect RD contained 2 μl of heat-killed mycobacterial DNA, a final concentration of 10 μl Hot Start Taq master mix (Qiagen), 0.3 μl primers RD9_FW, RD9_Rev, and RD9_IntRev (100 μM), and 7.1 μl sterile distilled water to a final volume of 20 μl. Thermal cycling was performed with a T-3000 Thermocycler Biometra Amplifier with an initial denaturation step of 15 min at 96 °C, 35 cycles of 30 s at 96 °C, 30 s at 55 °C, and 1 min at 72 °C, followed by a final elongation step of 10 min at 72 °C. PCR products were separated on a 1% agarose gel.

### Statistical analysis

Data were entered and cleared using EpiData version 3.1.Then they were exported to SPSS software version 20 for analysis.

Descriptive statistics were used for age, marital status, region of the participant, and to determine the prevalence of endometrial TB. The mean and standard deviation for age was also calculated. Crosstabs (kappa) was used for clinical diagnosis versus lab result, and for method evaluation respectively.

## Results

### Sociodemographic characteristics

As shown in Table 1, most of the study participants were in the age group of 34–40 years (mean age being 38.37±10 years) and married.

**Table 1:** Socio-demographic characteristics of study participants, Tikur Anbessa Specialized Hospital, Addis Ababa, Ethiopia

Socio-demographic characteristics	Range	Frequency (*N* = 152)	Percent
Age	20–26	16	10.5
	27–33	31	20.4
	34–40	54	35.5
	41–47	23	15.1
	48–54	19	12.5
	55–61	5	3.3
	62–68	2	1.3
	>/=69	2	1.3
Region	Addis Ababa	89	58.6
	Outside Addis Ababa	63	41.4
Marital status	Unmarried	29	19.1
	Married	111	73
	Divorced	8	5.3
	Widowed	4	2.6

Table 2 shows that the major complaint of the study participants was menstrual disturbance (73.7%) followed by chronic pelvic pain (59.2%). Endometrial TB was found by one of the three tests in 6.7% (6/90) of patients who were having chronic pelvic pain and in7.7% (5/65) of the women complaining of infertility. During recruitment, 29 women were investigated for infertility and 13.8% (4/29) were positive for endometrial TB. Only 71% of the participants were voluntarily screened for HIV and none of HIV positives had endometrial TB.

**Table 2:** Clinical profile of study participants, Tikur Anbessa specialized hospital, Addis Ababa, Ethiopia

Clinical background		Frequency (Total 152)	Relative frequency	Endometrial TB		Positive %
				Culture	IS1081-PCR only	
Primary infertility		43	28.3	1	3	7.7
Secondary infertility		22	14.5	1	0	
Investigated for infertility problem		29	19.1	2	2	13.8
Menses disturbance		112	73.7	1	2	2.8
	Menorrhagia	67	59.8	1	0	
	Amenorrhea	24	21.4	0	1	
	Oligomenorrhea	21	18.8	0	1	
Chronic Pelvic pain		90	59.2	4	2	6.7
HIV screen result	Positive	9	5.9	0	0	0.0
	Negative	99	65.1	2	2	4.0
	Undetermined	44	28.9	2	1	6.8
Anti TB before 2 years		13	8.5	0	0	0.0

### Prevalence of endometrial tuberculosis

The prevalence of endometrial TB was determined by culture with species confirmed by RD and IS1081-PCR for MTC. Consequently, 7(4.6%) were found to be members of the MTB complex (MTBC) byIS1081-PCR, of which only 4(2.6%) were culture positive and confirmed as *M. tuberculosis* by RD (Table 3).

**Table 3:** Detection of *M. tuberculosis* with different diagnostic methods, Tikur Anbessa Specialized Hospital, Addis Ababa, Ethiopia

Assays	No. of samples	Positive result (%)
PCR-IS1081	152	7(4.6)
Culture with AFB confirmed	152	5(3.3)
Culture with species confirmed	152	4(2.6)
Histo-pathology	152	2(1.3)
Combination of Culture and IS1081-PCR	152	7(4.6)
All tests	152	8(5.3)

### RD based species identification and multiplex PCR genus typing

**Fig. 2:**
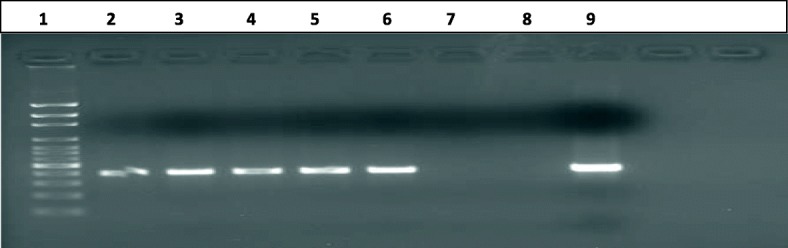
Isolates of mycobacterium characterized for species identification, using region of difference. Description; Lane 1 = ladder (100 bp), lane 2 through 6 = *M. tuberculosis* (396 bp), lane 7 = negative, lane 8 = negative control, lane 9 = positive control

After confirming the acid fastness of the isolates from the culture by Ziehl-Neelsen, further investigation was conducted to differentiate the species among the MTBC by the presence or absence of RD9. All culture positive AFB confirmed were *M.tuberculosis* (RD9 present) (Fig. 2), except one, which was later confirmed as not being a member of MTBC or even of the genus *Mycobacterium* by typing.

### Agreement between clinical criteria and culture and/or IS1081 PCR

The distribution of endometrial TB among suspected and non-suspected cases was analyzed and found to be prevalent in patients that had not been suggested by the physicians (71.4%, or 5/7) with the measure of agreement (kappa) 0.28 (Table 4) with both culture and/or IS1081-PCR and 75%of participants, who were culture positive only, were not suspected by physicians, with a kappa value of 0.17 (Table 5).

**Table 4:** Clinically suspected endometrial TB versus the rate of positivity by culture and/or IS1081-PCR

	Total (*n* = 152)	Culture + IS1081-PCR		Sensitivity %	Specificity %	PPV %	NPV %	kappa
Clinically Suspected for TB		Positive	Negative					
	Yes	2	4	28.6	97.2	33.3	96.6	0.28
	No	5	141

**Table 5:** Clinically suspected endometrial TB versus t rate of positivity by culture

	Total (*n* = 152)	Culture		Sensitivity %	Specificity %	PPV %	NPV %	kappa
Clinically Suspected for TB		Positive	Negative	25.0	96.6	16.7	97.9	0.174
	Yes	1	5					
	No	3	143					

### Evaluation of histopathological examination and IS1081-PCR with culture

**Fig. 3:**
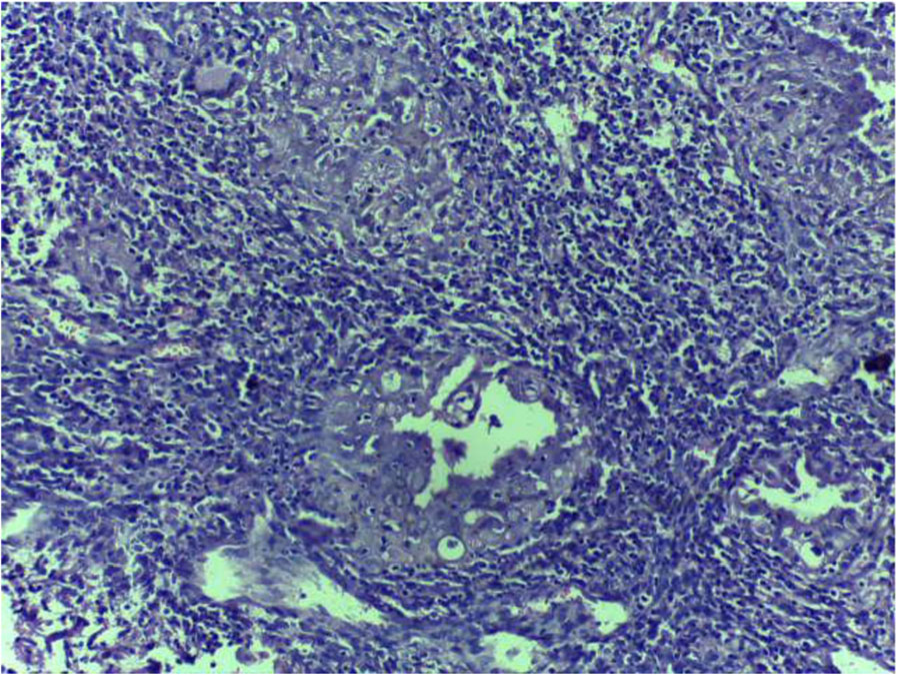
Endometrium with tuberculosis showing epitheloid cell aggregates and Langhan’s giant cells

The performance of histopathological examination and IS1081-PCR were evaluated against culture (Fig. 3). All culture positive samples were positive by IS1081-PCR, and one of two samples (50%) sample had a concordant result between histopathology and culture (Table 6).

**Table 6:** Comparison of IS1081-PCR and histopathology versus culture

Assays	Result Total (*n* = 152)	culture positive with species confirmed						Kappa value
		Positive	Negative	Sensitivity (%)	Specificity (%)	PPV (%)	NPV (%)	
IS1081-PCR	Positive	4	3	100	98	57	100	0.718
	Negative	0	145	25	50	50	98	0.321
Histology	Positive	1	1					
	Negative	3	147					

## Discussion

In this population of women who underwent endometrial biopsy for different gynecologic indications, it was found that the prevalence of endometrial TB was 4.6% by PCR, 2.6% by culture and 1.3% by histopathologic examination, showing a high prevalence rate similar to communities with a high overall TB prevalence rate [7–11, 15]. Since only endometrial samples were studied this will underestimate the prevalence and may not give the full picture about the occurrence of female genital TB. A similar study in India on general gynecological admissions identified 2.8% of GTB and 7.2% from infertile cases with a gynecological problem, with the help of a simplified TB algorithm [16]. The inclusion of mycobacteriological tests and molecular techniques could account for the higher report in our study though India is among the high TB burden countries.

Most studies in Africa and other Asian countries have given attention only to infertile women in order to assess the contribution of GTB to infertility, to understand the related clinical signs and symptoms and the consequence of treatment on conceiving. Our study identified endometrial TB in 6.7% of patients with chronic pelvic pain, 7.7% of women who had a problem of conception and in 13.8% of women who underwent endometrial biopsy as part of an infertility workup. A report from Tyerberg Hospital of South Africa, a country with a higher TB burden than Ethiopia, showed an incidence of 6% culture positive GTB in an infertile population [17]. A similar study conducted in India using hysterosalpinography [HSG] identified 6.3% GTB from all patients who underwent through the procedure which is in agreement with our study [18]. Similar to studies done in India, all positive cases were in the reproductive age group [19–21].

Of 152 women studied, 112 had one or another form of menstrual abnormality. Endometrial TB was detected in 2.8% of women with menstrual abnormality, similar to another study [22].

Literature reveals that *M. tuberculosis* is the most predominant species among the MTC species [23]. Similarly, in the present study, all isolates except one were confirmed as *M. tuberculosis* by deletion typing. One isolate, which was not in the genus *Mycobacterium*, could be an organism such as *Actinomyces*or *Nocardia,* that are known to cause pulmonary, cutaneous and sub-cutaneous diseases. They are acid fast by Ziehl Neelson staining, as is *Rhodococcus equi*, that causes pneumonia and is an acid fast cocco bacillus [24, 25]. Active GTB diagnosis was suggested by characteristic features of HSG, evidence of calcification/ complex adnoxal mass by scan, and the contact history of the patients. As a result, six patients were identified with possible GTB and two were positive for endometrial TB. One patient was positive by culture, IS1081-PCR and histology, but the other was detected only by IS1081-PCR. The overall kappa agreement, between clinical criteria and culture and/or IS1081 and culture only was found to be 0.28 and 0.17 respectively. Endometrial TB was more prevalent in patients not suspected of GTB (71.4%). This could be due to latent infection, when women are still asymptomatic, with low number of bacilli, and before structural damage to the tubes has taken place.

Rapid diagnosis and treatment of EPTB is required to lessen morbidity and mortality due to TB. It is crucial to have a sensitive and specific methodology for the diagnosis of TB in biopsies from genital organs in order to manage the disease easily. Different researchers have evaluated the performance of PCR compared to the conventional culture method in the diagnosis of TB from body fluid and biopsies. However, the primers used and the nature of the sample determines its sensitivity and specificity. In our findings, all culture positive samples (4/4) were also positive by IS1081-PCR with sensitivity, specificity, PPV and NPV of 100, 98, 57 and 100% respectively. Other researchers who evaluated IS1081-PCR from lymph node [26] and plural effusion [27] showed that the sensitivity was 91 and 84.6% respectively. Positive agreement between culture and IS1081-PCR was observed as 0.718, indicating good agreement. In contrast, Derese et al. reported 23.4% sensitivity on comparison of IS1081-PCR with standard culture from lymph node [28]. It is likely that the increased sensitivity of IS1081-PCR in our results reflects the small number of culture positive samples, good lysis of the mycobacterial cell wall and dissociation of DNA from particulate matter in the crude homogenate, allowing the recovery of supernatant after centrifugation and sufficient sample for extraction. The three (2%) culture negative but IS1081-PCR positive samples may be discrepant due to the presence of dead bacilli caused by storage or decontamination, and/or the absence or few bacteria in the culture sample.

Histopathology examination (HPE) is easy, quick and cheap. It is routinely used for the diagnosis of GTB, providing characteristic features of *M. tuberculosis* [29]. The primary aim of sample collection in this study was to examine HPE using haematoxylin and eosin staining for routine diagnosis of cancer and other diseases. Accordingly, two (1.3%) samples were positive for endometrial tuberculosis. One sample gave a concordant result with both culture and IS1081-PCR, but the other was negative by both methods. The agreement between culture and histopathology was found to be 0.321. A study in India [19] revealed that histopathology was more sensitive (6.9 Vs. 5.6), when compared against clinical criteria that indicated probable GTB, using HSG, laparascopy, ultrasound and other hematological tests. The possible reasons for the lower sensitivity of HPE in the present study could be the cyclical shedding of the endometrium that does not allow granuloma formation. Also, the sampled site may not represent the infected area.

The limitations of this study include the relatively small number of genital tuberculosis patients, which resulted in an inability to assess the sensitivity and accuracy of the different methods to diagnose female genital TB. Moreover, we included all patients for whom endometrial biopsy was requested for different indications and only endometrial samples were studied which might underestimate the prevalence and not give the full picture about the occurrence of female genital TB.

## Conclusions

The present finding of a 4.6% EPTB prevalence is alarming, especially its widespread distribution throughout the population. Similar to pulmonary and other extra-pulmonary TB, the causative agent of GTB was found to be *M. tuberculosis* in this study. Most patients positive for endometrial TB were not suspected by physical examination using clinical criteria. In the diagnosis of endometrial TB, IS1081-PCR was more sensitive and specific than histology or culture, even though culture is regarded as the gold standard. Therefore, using IS1081-PCR and/or culture for screening of GTB could greatly improve the sensitivity of diagnosing FGTB.

## Abbreviations

AAEC: AHRI/ALERT ethical committee
AHRI: Armauer Hansen Research Institute
ALIPB: Akililu lemma institute of patho-biology
DERC: Departmental ethical review committee
DMIP: Department of medical microbiology, immunology and parasitology
EPTB: extra-pulmonary TB
FGTB: Femalegenital tuberculosis
PCR: Polymerase chain reactio
PTB: Pulmonary tuberculosis
RD: Region of difference
TAHS: Tikur anbessa specialized hospital
TB: Tuberculosis
ZN: Ziehl-neelsen

## References

[1] Tripathy SN, Tripathy SN (2002). Infertility and pregnancy outcome in female genital tuberculosis. Int J Gynaecol Obstet.

[2] Weinberg AC, Boyd SD (1988). Short-course chemotherapy and role of surgery in adult and pediatric genito-urinary tuberculosis. Urology.

[3] Madhu N, Davinder P (2001). Genital tuberculosis: present scenario. JK Sciece.

[4] Bhanu NV, Singh UB, Chakraborty M, Suresh N, Arora J, Rana T (2005). Improved diagnostic value of PCR in the diagnosis of female genital tuberculosis leading to infertility. J Med Microbiol.

[5] Nogales-Ortiz F, Tarancón I, Nogales FF (1979). Pathology of female genital tuberculosis: a 31 year study of 1436 cases. Obstet Gynaecol.

[6] Parikh FR, Nadkarni SG, Kamat SA (1997). Genital tuberculosis a major pelvic factor causing infertility in Indian women. Fertile Steril.

[7] Jahromi NB, Parsanezhad ME, Ghane-Shirazi R (2001). Female genital tuberculosis and infertility. Int J Gynaecol Obstet.

[8] Saracoglu OF, Mungan T, Tanzer F (1992). Pelvic tuberculosis. Int J Gynaecol Obstet.

[9] de Vynck WE, Kruger TF, Joubert JJ, Scott F, van der Merwe JP, Hulme VA, Swart Y (1990). Genetal tuberculosis associated with female infertility in west cape. S Afr Med J.

[10] Varma TR (1991). Genital tuberculosis and subsequent fertility. Int J Gynecol Obstet.

[11] Sutherland AM (1983). The changing pattern of tuberculosis of the female genital tract. A thirty-year survey. Arch Gynecol.

[12] Kubica GP, Dye WE, Cohn ML (1964). Sputum digestion and decontamination with N-acetyl-L-cysteine-sodium hydroxide for culture of mycobacteria. Am Rev Respir Dis.

[13] Truffot-Pernot C, Véziris N, Sougakoff W (2006). Modern diagnosis of tuberculosis. Presse Med.

[14] Brosch R, Gordon SV, Marmiesse M, Brodin P, Buchrieser C, Eiglmeier K (2002). A new evolutionary scenario for the *Mycobacterium tuberculosis* complex. Proc Natl Acad Sci U S A.

[15] Abebe M, Kidane D, Kirose K, Harboe M. Female genital tuberculosis in Ethiopia. Int J Gynaecol Obstet. 2004;84:241–6. 10.1016/j.ijgo.2003.11.002 15001372

[16] Jindal UN (2006). An algorithmic approach to female genital tuberculosis causing infertility. Int J Tuberc Lung Dis.

[17] Margolis K, Wranz P, Kruger TF, Joubert JJ, Odendaal H (1992). J. Genital tuberculosis at Tygerberg hospital – prevalence, clinical presentation and diagnosis. S Afr Med J.

[18] Chavhan GB, Hiva P, Rathod K, Zacharia TT, Chawla A, Badhe P (2004). Female genital tuberculosis: Hysterosalpingographic appearances. Br J Radiol.

[19] Thangappah RBP, Paramasivan CN, Narayanan S (2011). Evaluating PCR, culture & histopathology in the diagnosis of female genital tuberculosis. Indian J Med Res.

[20] Kohli MD, Nambam B, Trivedi SS, SB L, Arora S, Jain A (2011). PCR-based evaluation of tuberculous endometritis in infertile women of North India. J Reprod Infertil.

[21] Kulshrestha V, Kriplani A, Agarwal N, Singh UB, Rana T (2011). Genital tuberculosis among infertile women and fertility outcome after antitubercular therapy. Int J Gynaecol Obstet.

[22] Othieno E, Odida M, Gemagaine G, Okwi A, Bimenya GS, Wandabwa J (2008). Female genital tuberculosis in Uganda. AJABS.

[23] Kallenius G, Koivula T, Ghebremichael S, Hoffner SE, Norberg R, Svensson E (1999). Evolution and clonal traits of Mycobacterium tuberculosis complex in Guinea-Bissau. J Clin Microbiol.

[24] Halpern M, Castineiras M, Souza S, Hofer E, Martins S (1994). Cavitary pneumonia caused by Rhodococcus equi in patient with AIDS. Int Conf AIDS.

[25] Taylor GM, Murphy E, Hopkins R, Rutland P, Chistov Y (2007). First report of Mycobacterium bovis DNA in human remains from the Iron age. Microbiology.

[26] Taylor GM, Worth DR, Palmer S, Jahans K, Hewinson RG (2007). Rapid detection of Mycobacterium bovis DNA in cattle lymph nodes with visible lesions using PCR. BMC Vet Res.

[27] Bahador A, Etemadi H, Kazemi B, Ghorbanzadeh R, Nakhjavan FA, Nejad ZA (2005). Performance assessment of IS1081-PCR for direct detection of tuberculous pleural effusion: compared to rpoB-PCR. Res J Agric Biol Sci.

[28] Derese Y, Hailu E, Assefa T, Bekele Y, Mihret A, Aseffa A (2012). Comparison of PCR with standard culture of fine needle aspiration samples in the diagnosis of tuberculosis lymphadenitis. J Infect Dev Ctries.

[29] Sathe AV, Vaidya PR, Deshmukh MA, Motashaw ND (1979). Genital tuberculosis in an endocrine clinic. J Obstet Gynaecol India.

